# Study protocol of EMPOWER Participatory Action Research (EMPOWER-PAR): a pragmatic cluster randomised controlled trial of multifaceted chronic disease management strategies to improve diabetes and hypertension outcomes in primary care

**DOI:** 10.1186/1471-2296-15-151

**Published:** 2014-09-13

**Authors:** Anis S Ramli, Sharmila Lakshmanan, Jamaiyah Haniff, Sharmini Selvarajah, Seng F Tong, Mohamad-Adam Bujang, Suraya Abdul-Razak, Asrul A Shafie, Verna KM Lee, Thuhairah H Abdul-Rahman, Maryam H Daud, Kien K Ng, Farnaza Ariffin, Hasidah Abdul-Hamid, Md-Yasin Mazapuspavina, Nafiza Mat-Nasir, Maizatullifah Miskan, Jaya P Stanley-Ponniah, Mastura Ismail, Chun W Chan, Yong R Abdul-Rahman, Boon-How Chew, Wilson HH Low

**Affiliations:** Primary Care Medicine Discipline, Faculty of Medicine, Universiti Teknologi MARA, Selayang Campus, 68100 Batu Caves, Selangor Malaysia; Clinical Epidemiology Unit, National Clinical Research Centre, Ministry of Health, Kuala Lumpur, Malaysia; Department of Social and Preventive Medicine, Faculty of Medicine, University of Malaya, Kuala Lumpur, Malaysia; Department of Family Medicine, Faculty of Medicine, Universiti Kebangsaan Malaysia, Kuala Lumpur, Malaysia; School of Pharmaceutical Sciences, Universiti Sains Malaysia, Kragujevac, Penang Malaysia; Department of Family Medicine, Faculty of Medicine, International Medical University, Bukit Jalil, Kuala Lumpur, Malaysia; Centre for Pathology and Diagnostic Research Laboratory, Faculty of Medicine, Universiti Teknologi MARA, Sungai Buloh, Selangor Malaysia; Klinik Kesihatan Seremban 2, Kragujevac, Negeri Sembilan Malaysia; Family Medicine Discipline, Faculty of Medicine, Cyberjaya University College of Medical Sciences, Cyberjaya, Selangor Malaysia; Department of Family Medicine, Faculty of Medicine and Health Sciences, Universiti Putra Malaysia, Serdang, Selangor Malaysia; Azmi Burhani Consulting Sdn. Bhd, Petaling Jaya, Selangor Malaysia

**Keywords:** Chronic disease management, Chronic care model, Multifaceted intervention, Primary care, Type 2 diabetes mellitus, Hypertension

## Abstract

**Background:**

Chronic disease management presents enormous challenges to the primary care workforce because of the rising epidemic of cardiovascular risk factors. The chronic care model was proven effective in improving chronic disease outcomes in developed countries, but there is little evidence of its effectiveness in developing countries. The aim of this study was to evaluate the effectiveness of the EMPOWER-PAR intervention (multifaceted chronic disease management strategies based on the chronic care model) in improving outcomes for type 2 diabetes mellitus and hypertension using readily available resources in the Malaysian public primary care setting. This paper presents the study protocol.

**Methods/Design:**

A pragmatic cluster randomised controlled trial using participatory action research is underway in 10 public primary care clinics in Selangor and Kuala Lumpur, Malaysia. Five clinics were randomly selected to provide the EMPOWER-PAR intervention for 1 year and another five clinics continued with usual care. Each clinic consecutively recruits type 2 diabetes mellitus and hypertension patients fulfilling the inclusion and exclusion criteria over a 2-week period. The EMPOWER-PAR intervention consists of creating/strengthening a multidisciplinary chronic disease management team, training the team to use the Global Cardiovascular Risks Self-Management Booklet to support patient care and reinforcing the use of relevant clinical practice guidelines for management and prescribing. For type 2 diabetes mellitus, the primary outcome is the change in the proportion of patients achieving HbA1c < 6.5%. For hypertension without type 2 diabetes mellitus, the primary outcome is the change in the proportion of patients achieving blood pressure < 140/90 mmHg. Secondary outcomes include the proportion of patients achieving targets for serum lipid profile, body mass index and waist circumference. Other outcome measures include medication adherence levels, process of care and prescribing patterns. Patients’ assessment of their chronic disease care and providers’ perceptions, attitudes and perceived barriers in care delivery and cost-effectiveness of the intervention are also evaluated.

**Discussion:**

Results from this study will provide objective evidence of the effectiveness and cost-effectiveness of a multifaceted intervention based on the chronic care model in resource-constrained public primary care settings. The evidence should instigate crucial primary care system change in Malaysia.

**Trial Registration:**

ClinicalTrials.gov NCT01545401

## Background

Chronic diseases, led by cardiovascular diseases (CVD)—mainly ischaemic heart disease and stroke [1, 2], are the largest cause of morbidity and mortality in the world. The majority of the chronic disease burden occurs in the low- and middle-income countries [1–3]. In Malaysia, chronic conditions were responsible for 69% of the total burden of disease as measured in disability-adjusted life years (DALYs) [4]. Chronic diseases accounted for 71% of all deaths, of which 30% were caused by CVD [5]. In addition, there is a trend towards younger age at first myocardial infarction [6] and higher CVD mortality in Malaysia compared to developed countries [5]. This disturbing phenomenon is caused by the rising epidemic of cardiovascular (CV) risk factors such as hypertension (HPT) and type 2 diabetes mellitus (T2DM) over the past 30 years [1]. The National Health and Morbidity Survey 2011 showed that the prevalence of HPT and T2DM among Malaysian adults had reached epidemic proportion at 32.7% and 15.2%, respectively [7].

In Malaysia, the majority of common chronic diseases are managed in primary care [8]. This presents an enormous challenge to the primary care workforce, because resources are often limited and are further divided into the dual system of public and private primary care sectors. There is an imbalanced distribution of resources between the public and private sectors, where the number of private clinics outnumbered the public clinics by 6.3 to 1 in 2008–2009 [8]. The public sector is served by 0.52 doctors per 10,000 population, whereas the private sector is served by 2.37 doctors per 10,000 population [8]. Private primary care doctors often work in independent practices deficient of allied health support, whereas public primary care clinics are staffed by trained family physicians known as family medicine specialists (FMS), medical officers without postgraduate qualifications, paramedical practitioners known as assistant medical officers, nurses, pharmacists and dieticians/nutritionists [8]. In terms of funding, the public primary care sector is subsidised by the government and patients pay a minimal sum for treatment. In the private sector, costs are largely borne by the patients, their employers or insurance companies [9]. Therefore, it is often too expensive for patients with multiple chronic conditions to bear the out-of-pocket costs in the private sector [9]. As a result, the over-subsidised and under-staffed public primary care clinics are overburdened, providing care to a larger proportion of patients with chronic conditions (33.7/100 encounters) compared with the private primary care clinics (5.6/100 encounters) [10]. Although public primary care clinics have access to allied health care personnel, the effective implementation of multidisciplinary management of chronic diseases is often hampered by high turnover and shortages of staff, high patient load and time constraints [9]. Overall, the Malaysian primary care system is still orientated towards the care of acute, episodic illnesses [9].

The strain and limitation in the Malaysian primary care system may have contributed to the suboptimal management and control of chronic diseases. A nationwide audit of diabetes care and management conducted in public primary care clinics showed that only 18.1% of T2DM patients had attained an HbA1c of < 6.5% [11]. A study in six public primary care clinics revealed blood pressure (BP) control was achieved in 24.3% of HPT patients with T2DM and 60.1% of HPT patients without T2DM [12]. Similarly, a study looking at the process of care and the choice of antihypertensive medications in public and private primary care clinics in Malaysia revealed that 21% of prescription practices were less than optimal in both sectors [13]. A lipid control study of diabetics revealed that the target low density lipoprotein cholesterol (LDL-C) of ≤ 2.6 mmol/L was achieved in only 22% of diabetic patients attending a public primary care clinic in Sarawak, Malaysia [14].

Poor management of chronic diseases in primary care can lead to the massive burden of treating complications in secondary care, as well as burdening patients and their families because of morbidity and premature deaths, and burdening the country because of the premature loss of human capital [3]. Improving the prevention and management of chronic diseases in primary care should therefore be a priority for low- and middle-income countries [15–17]. However, when resources are limited, priorities should be given to the most cost-effective strategy that can produce swift changes [18]. An integrated and multifaceted approach to the management of chronic diseases in primary care has been shown to be cost-effective [19]. Because the majority of the chronic disease burden is caused by CVD, the World Health Organization (WHO) advocates integrated and multidisciplinary intervention in primary care, targeting those with multiple CV risk factors [20].

However, it is vital to recognise that chronic diseases management (CDM) is fundamentally different from acute care, demanding a complex health-systems response that needs to be sustained across the continuum of care [16]. CDM involves opportunistic case finding for assessing risk factors, detection of early disease and identification of high-risk patients, as well as pharmacological and psychosocial interventions. It often requires a stepped-care approach involving long-term follow-up with regular monitoring and promotion of adherence to treatment. To meet the challenge of chronic diseases, primary care systems will have to be redesigned and strengthened substantially [16, 17, 19]. Fundamental changes are needed in the way care is structured and delivered [16].

In view of the growing needs to re-orientate the health system, the WHO proposed a model for change, namely the Innovative Care for Chronic Conditions (ICCC) Framework [16]. This framework took its reference from the chronic care model (CCM), an evidence-based model developed by Wagner and colleagues [21–23]. The CCM offers a solution that shifts the paradigm from the acute care model to a comprehensive, patient-centred model of care, which integrates multifaceted elements to improve the quality of chronic disease outcomes [21–23]. It argues that optimal CDM is achieved when a well-coordinated, proactive health care team interacts productively with an empowered and motivated patient [21–23]. Evidence has shown that successful implementations of the CCM have been framed within pragmatic environments and have used collaborative and participatory approaches aiming at empowering health care providers to improve clinical practice [24]. Primary care providers should therefore be empowered with knowledge and skills and be given the autonomy to make the choice of actions within their constraints to improve their patients’ health outcomes [25].

Evidence from developed countries has shown that primary care practice redesigned in accordance with the CCM improves the quality of care and outcomes for patients with various chronic conditions [26–28]. This model has greatly influenced the reorganisation of chronic disease care in many developed countries such as Australia, the United Kingdom and the United States of America [28–31]. Experiences from previous studies have also shown that this innovative model of care results in higher short-term costs owing to the increase in resources required to change the current model of care [32]. However, recent evidence of this model’s effectiveness in reducing HbA1C and risk of end-stage renal disease could potentially translate to better quality-adjusted life years (QALY), making it cost-effective in the long run [33]. Furthermore, because the CCM relies on the innovative use of existing health care resources, the implementation of this model of care would be different from one country to another; hence, the results of cost-effectiveness analyses of such initiatives should be context- and country-specific [33].

Evidence on the effectiveness of the CCM in developing countries is also emerging [34, 35]. The CORFIS study, a pragmatic, non-randomised controlled trial involving 70 private primary care clinics in Malaysia, showed that the proportion of hypertensive patients who achieved target BP after 6 months was significantly higher in the CORFIS arm (69.6%) as compared with the control arm (57.6%), P = 0.008 [35]. The CORFIS intervention was designed based on five of the six CCM elements [35]. However, further research is needed to evaluate the effectiveness and cost-effectiveness of this intervention in the Malaysian public primary care setting, where a larger proportion of patients with chronic conditions are receiving care and where limited resources are often stretched thin. Therefore, the main aim of this study was to evaluate the effectiveness of the EMPOWER-PAR intervention (multifaceted CDM strategies designed based on the CCM) in improving clinical outcomes for patients with T2DM and/or HPT using existing health care resources in the Malaysian public primary care setting. This paper describes the design of the trial, the details of the EMPOWER-PAR intervention and its underpinning conceptual framework.

### Hypotheses

The primary hypothesis for T2DM patients was that the proportion achieving the target HbA1c of < 6.5% would improve with the EMPOWER-PAR intervention. The primary hypothesis for HPT patients without T2DM was that the proportion achieving the target BP of < 140/90 mmHg would improve with the EMPOWER-PAR intervention.

The secondary hypothesis for T2DM patients was that the proportion achieving the target BP of ≤ 130/80 mmHg would improve with the EMPOWER-PAR intervention. The secondary hypotheses for both groups of patients (T2DM and HPT without T2DM) were that the proportions achieving target fasting serum lipid, body mass index (BMI) and waist circumference (WC) would improve with the intervention. Medication adherence levels and patients’ perceptions of their experience of chronic disease care were also expected to improve with the intervention. Secondary hypotheses were also made at the primary care providers’ level in terms of the process of care and prescribing pattern, which were expected to improve with the intervention. Cost-effectiveness and primary care providers’ perceptions, attitudes, experiences and perceived barriers in implementing the intervention were also explored.

## Methods/Design

This is a pragmatic cluster randomised parallel controlled trial currently being conducted in 10 public primary care clinics in two states in Malaysia—Wilayah Persekutuan Kuala Lumpur (WPKL) and Selangor (SEL). The overall duration of the study is two years, and the duration of intervention is one year. Blinding was not possible due to the nature and complexity of the intervention. The study protocol is registered with clinicaltrial.gov (NCT01545401), and the reporting of this paper has been done in accordance with the CONSORT Statements [36–38].

The cluster randomised trial design was used because of its strengths in evaluating educational and interventional programmes in health care units [38]. The pragmatic study design was chosen to maximise external validity to ensure that the results can be generalised to the public primary care system in Malaysia [39]. The participatory action research (PAR) approach [25, 40] was adopted in the design and implementation of the EMPOWER-PAR intervention to ensure that the primary care providers involved in this study are empowered to choose between actions within their constraints to improve their patients’ health outcomes.

### Site recruitment

#### Site eligibility

All 34 clinics led by FMS in SEL and WPKL were invited to participate in the study and were given the site feasibility assessment form.

To be eligible, the clinics were required to satisfy all of the following criteria:

1. have a minimum of 500 T2DM patients and 500 HPT patients in the registry
2. have an FMS who is keen to participate in the study and willing to lead the implementation of the intervention components in the clinic
3. have the minimum capacity to implement the obligatory components of the EMPOWER-PAR intervention
4. be located within 70 km of the central laboratory (as the blood samples were transported back to the centre for analysis)

#### Site selection

A site feasibility assessment was conducted to identify eligible clinics. Out of the 34 sites, only 20 fulfilled the eligibility criteria to enter the study. These 20 clinics were then matched into 10 pairs according to their geographical locations, staffing and workload. Geographical location was divided into urban and suburban areas. An urban area was defined as an area located within a major city, while a suburban area was defined as the surrounding area located within commuting distance to a major city. Staffing was defined as the number of doctors and allied health personnel (FMS, medical officers, assistant medical officers, staff nurses, dieticians/nutritionists and pharmacists) working in the clinic. Workload was defined as the average number of patients seen in the clinic per day.

Multistage randomisation was performed using computer-generated tables. The first stage was to randomly select five of the 10 pairs to be included into the study. The second stage was to randomly allocate the clinics into intervention and control arms. Table 1 summarises the characteristics of the randomly selected clinics.

**Table 1:** **Characteristics of EMPOWER-PAR Intervention Clinics and Control Clinics**

Clinic characteristics		Geographical location	Workload (average number of patients seen in the clinic per day)	Staffing (number of doctors and allied health personnel)
Pair no. 1	**Intervention**	**Urban**	**900**	**30**
	Control	Urban	900	28
Pair no. 2	**Intervention**	**Urban**	**600**	**27**
	Control	Urban	650	29
Pair no. 3	**Intervention**	**Urban**	**550**	**32**
	Control	Urban	500	33
Pair no. 4	**Intervention**	**Sub-urban**	**500**	**22**
	Control	Sub-urban	500	20
Pair no. 5	**Intervention**	**Sub-urban**	**350**	**21**
	Control	Sub-urban	400	19

### Patient recruitment

This study recruited consecutive T2DM and/or HPT patients who attend the selected clinic within the 2-week recruitment period. These patients were given the patient information sheet in the waiting area. Informed consent forms were obtained from those willing to participate. Screening was conducted to identify eligible participants based on the inclusion and exclusion criteria. Eligible patients were then enrolled in the study.

#### Inclusion criteria

Males and females aged ≥ 18 years who were:

1. diagnosed with T2DM and/or HPT
2. seen at least once in the last year at the primary care clinic for the above condition(s)

#### Exclusion criteria

**Figure 1:**
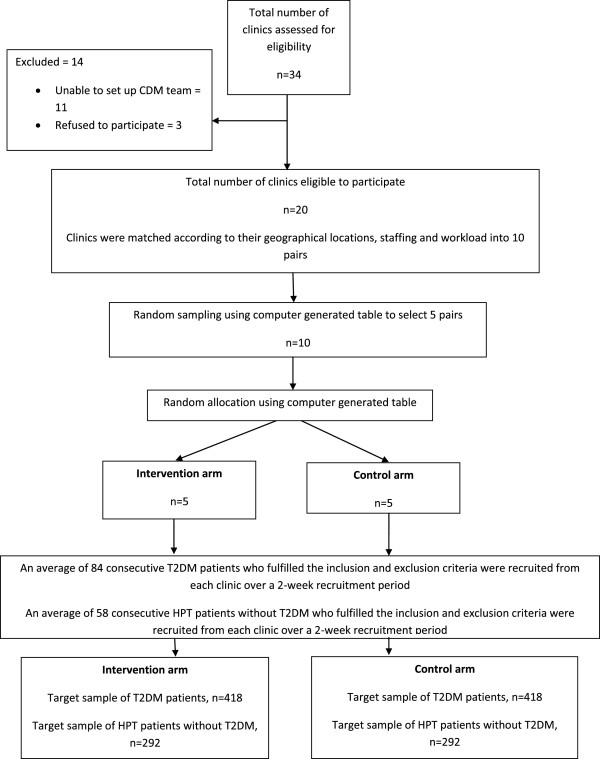
**EMPOWER-PAR Trial Profile: Enrolment of Public Primary Care Clinics and Recruitment of Patients.**

1. type 1 diabetes mellitus
2. receiving renal dialysis
3. presenting with severe HPT (Systolic BP > 180 mmHg and/or Diastolic BP > 110 mmHg)
4. diagnosed with conditions resulting in secondary hypertension
5. diagnosed with circulatory disorders requiring referral to secondary care over the last year (e.g. unstable angina, heart attack, stroke, transient ischaemic attacks)
6. receiving shared care between primary and secondary care for complications of T2DM and/or HPT
7. pregnancy
8. enrolled in another studyAll patients in the intervention arm were required to be seen at least twice by the CDM team of each clinic during the 1-year intervention period. Those who did not comply were considered as lost to follow-up. There was no limit to the number of clinic visits a patient was allowed to make in either arm during the course of the study. Figure 1 shows the EMPOWER-PAR Trial Profile outlining the enrolment of public primary care clinics and the recruitment of patients.

### The EMPOWER-PAR intervention

The study intervention, referred to as the EMPOWER-PAR intervention, was developed in accordance with Medical Research Council guidance on developing and evaluating complex interventions to improve health outcomes [41] and was implemented at the primary care clinics for a period of one year. The underlying framework in developing the complex intervention for EMPOWER-PAR was based on six interrelated elements of the CCM [21–23]. The six elements are 1) organisation of health care (i.e. providing leadership and minimising barriers to care), 2) self-management support (i.e. facilitating skills-based learning and patient empowerment), 3) decision support (i.e. providing guidance for implementing evidence-based care), 4) delivery system design (i.e. coordinating care processes), 5) clinical information systems (i.e. tracking progress through reporting outcomes to patients and providers) and 6) community resources and policies (i.e. sustaining care by using community-based resources) [21–23].

Three obligatory components that define the EMPOWER-PAR intervention were developed based on four interrelated elements of the CCM (organisation of health care, delivery system design, self-management support and decision support). These intervention components utilise readily available and existing resources in the Malaysian public primary care setting:

I. Creating/Strengthening a CDM team—a multidisciplinary team led by FMS to improve coordination of care for T2DM and/or HPT and co-existing CV risk factors
II. Utilising the Global CV Risks Self-Management Booklet to support patients self-management
III. Utilising the Clinical Practice Guidelines (CPG) for T2DM [42] and HPT [43] to assist with management and prescribing

Two optional components of the EMPOWER-PAR intervention were developed based on the other 2 CCM elements (clinical information system and community resources):

I. Utilising clinical information system and conducting clinical audits to track progress through reporting outcomes to patients and providers
II. Utilising community resources to support and sustain care

### Evidence on the chronic care model and participatory action research approach as the underpinning conceptual frameworks for the EMPOWER-PAR intervention

The CCM has been identified by global health policy experts as the most advanced in terms of conceptualisation and design, and in the range of evidence supporting it [16, 34]. Several systematic reviews [26–28, 44] and a meta-analysis [45] evaluating the impacts of the CCM have demonstrated the effectiveness of this model in a variety of settings, pathologies and target groups. Evidence has also shown that system approaches addressing even one of the components have been helpful in improving quality [27]. Some researchers have even attempted to identify which of the individual CCM elements were the most effective, despite the difficulty of disentangling the multifaceted elements. Organisation of health care that involves providing leadership and removing barriers to care is one the most important elements of the CCM [46–48]. Evidence has shown that measurable improvement in the care of patients with chronic conditions will only occur if system leaders make it a priority and provide the leadership, incentives and resources necessary to make improvements happen [46]. Delivery system design and self-management support have also emerged as powerful interventions [47, 48]. Decision support that integrates locally relevant evidence-based CPG into the fabric of patient care is fundamental to putting evidence into practice in managing chronic diseases [49].

In view of this evidence, the three obligatory components of the EMPOWER-PAR intervention were designed based on four interrelated CCM elements, namely organisation of health care, delivery system design, self-management support and decision support. These elements were chosen because of their individual strength in improving chronic care quality. In EMPOWER-PAR, the organisation of health care and delivery system design was delivered in the form of creating or strengthening the multidisciplinary CDM team and improving the coordination of care. The FMS had to be willing to lead the CDM team as well as the implementation of the intervention to be included in the study. Being the most clinically qualified member of the team, the FMS was ideally suited to take up the leadership role. The CDM team consists of multidisciplinary allied health personnel, and their roles are pivotal in improving the clinical outcomes, given the complexity of managing chronic conditions [50]. Evidence has shown that multidisciplinary interventions have resulted in significant improvement in glycaemic and BP control [51–53]. Delivery system design also involves training the CDM team to improve the coordination of care, particularly in the skills to develop and maintain clinic-based registries, an appointment system, reminder mechanisms and defaulter tracing. This element is central in improving the process of care for patients with chronic diseases whom require long-term follow-up, regular monitoring and promotion of adherence to treatment [50].

Self-management support was delivered to patients using the Global CV Risks Self-Management Booklet. The CDM team were trained in patient-centred communication and counselling skills to empower the patients to self-manage. There is ample evidence to show that application of these skills in delivering self-management support to patients improved glycaemic and BP control compared to usual care, beyond the benefits afforded by medications alone [54–56].

Decision support was delivered by training the CDM team to utilise the CPG on T2DM [42] and HPT [43] to improve management and prescribing. Evidence from other studies suggested that adherence to evidence-based prescribing guidelines for hypertension resulted in substantial savings in prescription costs [57]. Although the implementation of the other two CCM elements (clinical information system and community resources) as part of the EMPOWER-PAR intervention was optional, evidence supporting their role in CDM is also robust [58, 59].

Despite strong evidence supporting the individual elements of the CCM, there is a paucity of literature regarding implementation of the entire CCM as a multifaceted intervention, especially in developing countries. With the exception of the CORFIS study [35], previous studies implementing the entire CCM as multifaceted intervention have been conducted in developed countries [60, 61]. Similar to CORFIS, the EMPOWER-PAR is not designed to differentiate the effectiveness of individual CCM elements in its multifaceted intervention.

Successful CCM implementations have also been shown to work best within the pragmatic environment using a collaborative approach and coaching initiatives aiming at empowering health care providers to improve clinical practice [24]. External guidance and the participation of multidisciplinary health care team members during the process have been shown to be the contributing factors towards successful implementation and real transformation [24]. However, contextual factors within different clinic settings may influence the ability of teams to make and sustain changes in care. Although the selected clinics have great similarities, the existing system of care for chronic diseases may differ substantially. Each clinic may already have a pre-existing chronic disease management system, and these are at different stages of development depending on the experience of the FMS in charge and their duration of service at the clinic. Clinics with an experienced and longer serving FMS may be more advanced in their system development for chronic disease care. Furthermore, there may be local challenges resulting in the different stages of the development. The challenges include shortages or high turnover of medical staff and allied health personnel, limited clinic space, high patient load and time constraints. These differences demand different levels of intervention and expertise for improvement, and imposing a rigid intervention program is impractical and inappropriate. Hence, this study did not impose a strict protocol, and some flexibility on how the intervention program was implemented was allowed in line with the needs and constraints of the clinics.

The PAR approach [25, 40] was therefore adopted in designing and implementing the EMPOWER-PAR intervention. This process requires active participation of the CDM team from each clinic to design, propose and implement the intervention program based on the four CCM elements that define the intervention. In PAR, the researchers attempt to democratise the research process [25]. The iteration of reflection and self-analysis of the intervention, together with the power sharing in the research process are the main characteristics of PAR [25, 40]. Participants who were passive players in the beginning would eventually become active players [25]. The PAR component ensured that the primary care providers involved in this study were empowered to make the choice of actions within their constraints to improve their patients’ health outcomes. The process of PAR allows the primary care providers to have increased autonomy to determine how best to improve the quality of their patient care [25].

### Implementation process of the intervention

The intervention clinics received the EMPOWER-PAR intervention package, which consists of CDM workshops, intervention tools, facilitation and support for a period of one year.

The implementation process was conducted in 3 phases:

- Phase 1: Formation and training of the CDM team
- Phase 2: Distribution of the intervention tools

- Global CV Risks Self-Management Booklets
- CPG to assist with clinical decision making

Phase 3: Facilitation and support to implement the intervention

#### Phase 1: Formation and training of the CDM team

Each intervention clinic was required to identify at least five CDM team members, including the FMS, medical officer, medical assistant, nurse, pharmacist and dietician/nutritionist. The team was required to be led by the FMS and was trained in the CDM Workshops.

The CDM workshops consisted of three workshop series of 1.5 days each, and the objectives and contents were designed based on the six interrelated elements of the CCM. These workshop series were piloted in an urban public primary care clinic in Negri Sembilan. Written and verbal feedback regarding the workshop was obtained from the participants. The workshop’s objectives, content and teaching and learning methods were then refined accordingly. Table 2 summarises the finalised CDM workshop’s objectives, content and teaching and learning methods. During the workshop, the CDM team from each clinic was divided into small groups to discuss the challenges and brainstorm on the details of how best to deliver the intervention within their limitations and constraints. At the end of the workshop series, the CDM team from each clinic prepared a proposed intervention plan describing the steps needed to achieve their goals within a set timeline. The agreed plan of action took into account their constraints and how to overcome those barriers.

**Table 2:** **EMPOWER-PAR CDM Workshops’ Objectives, Contents and Teaching–Learning Methods**

Workshops	CCM elements covered	Objectives	Contents	Teaching-learning methods
Workshop 1	• Organisation of health care (providing leadership and removing barriers to care)	At the end of this workshop, the participants should be able to:	1. Introduction to CDM and CCM	• Lecture
	• Delivery system design (coordinating care processes)	• Discuss the concept and principles of CDM & the CCM	2. Redesigning delivery of care for chronic conditions	• Small group hands-on sessions
		• Discuss the need to coordinate care for chronic conditions using multidisciplinary care team	3. Building a multidisciplinary CDM Team	• Group presentation
		• Define roles and responsibilities of the team members	• Defining roles and responsibilities	
		• Formulate a plan on how to re-design the delivery of chronic care in your own practice setting	• Identifying barriers and resolving potential conflicts	
		• Formulate a plan on how to improve care coordination	• Improving care coordination	
			4. Delivery system re-design to improve care coordination	
			• Developing clinic-based registries	
			• Creating appointment system, reminder mechanisms and defaulter tracing	
Workshop 2	• Self-management support (facilitating of skills-based learning and patient empowerment)	At the end of this workshop, the participants should be able to:	1. Introduction to self management support	• Lectures
		• Discuss the concept and principles of self-management support	2. Patient-centred communication:	• Small group hands-on sessions
		• Demonstrate patient-centred consultation to support patients’ self-management	• Building relationship and partnership	• Consultation practice of various clinical scenarios using simulated patients and the Global CV Risks Self-Management Booklet as a tool
		• Guide patients to make informed decision	• Shared decision making	
		• Motivate patients to change their behaviour	3. Building Relationship	
		• Utilise the Global CV Risks Self-Management Booklet to empower patients	• Gathering clinical information & patient experience	
			• Exploring ideas, concerns and expectations	
			• Engaging patient	
			4. Sharing information and goal setting	
			• Providing sufficient information	
			• Explaining in simple language	
			• Assessing understanding	
			• Goal setting	
			5. Reaching agreement in management plan	
			• Involving patient in decision making process	
			• Reaching agreement	
			6. Motivating patients to change	
			• Motivating patients to change their lifestyle	
			• Achieving adherence to therapy	
			• Self-monitoring of blood pressure and blood glucose	
			• Supporting patients with self management tools	
Workshop 3	• Decision support (providing guidance for implementing evidence-based care)	At the end of this workshop, the participants should be able to:	1. Introduction to evidence-based care and decision support	• Lectures
	• Clinical information systems (tracking progress through reporting outcomes to patients and providers)	• Discuss the importance of evidence-based care	2. Implementing CPG	• Small group hands-on sessions
	• Community resources and policies (sustaining care by using community-based resources)	• Identify potential solutions to improve CPG implementation in primary care clinics	• Identifying facilitators for change and possible solutions	
		• Utilize the T2DM and HPT CPG to aid management and prescribing.	• Using CPG in daily clinical practice	
		• Formulate a plan on how to improve the clinical information system (CIS)	3. Improving CIS and designing a clinical audit project	
		• Discuss the importance of Clinical Audit in improving quality of chronic disease management	• Identifying areas needing improvement	
			• Sampling frame and sample sizes	
			• Sampling methods	
			• Activity charts	
		• Design a Clinical Audit Project	• Criteria and standards	
		• Recommend remedial actions to improve chronic care quality	• Preparing data collection format	
			• Data analysis and interpretation of results	
			• Remedial action plan and	
		• Discuss the importance of community resources	• Implementation	• Group presentation
			• Completion of the audit cycle	
			• Distributing tasks among team members	
			4. Community Resources	
			• Identifying available resources in your community	
			• Developing collaborative partnership with NGO’s and community leaders	

Apart from the CDM workshop, an intervention review workshop was conducted 6 months after the commencement of intervention. In this workshop, the intervention clinics were invited for a 1-day session to share their experiences and challenges in implementing the intervention. The main objective of this workshop was to allow interactions among the participating clinics and solve any arising problems.

#### Phase 2: Distribution of the intervention tools

The intervention tools consist of the i) Malaysian CPG and the Quick References (QR) on the Management of T2DM [42] and HPT [43] to assist health care providers in clinical decision making; and ii) the Global CV Risks Self-Management Booklet to support patients’ empowerment and self-management skills in managing their T2DM and/or HPT.

The CPG and QR were distributed to the CDM team during the third CDM workshop, where they were trained on how to recognise the facilitators and barriers for CPG utilisation and on how to find solutions to improve CPG utilisation in their daily clinical practice. CPG training using interactive case discussion was also conducted during the intervention review workshop.

The Global CV Risks Self-Management Booklet was designed by the investigators to suit local needs. It was a 49-page booklet that is divided into four sections. The first section contained information to empower patients with knowledge regarding their global CV risks (including T2DM, HPT, dyslipidaemia, overweight/obesity, cigarette smoking and family history of premature CVD), presence/absence of complications, CV risk stratification and treatment targets. The second section contained on-going information of the patient’s routine physical examination findings and investigation results. The third section contained instructions on how to perform self-monitoring of blood glucose and home BP monitoring and dedicated pages where patients could record their own readings. The fourth section contained the patient’s medications record. Health care providers were trained on how to use the booklet to support patients in self-management of T2DM and/or HPT during the second CDM workshop. The information and instructions were written in English and Malay. At least 5000 booklets were distributed to each intervention clinic, and this booklet was given to all T2DM and/or HPT patients registered in the clinic.

#### Phase 3: Facilitation and support to implement the intervention

Each clinic was assigned two facilitators to coach the CDM team, facilitate and guide the implementation of intervention and provide feedback on their performance. Facilitators were the investigators, all of whom are qualified FMS or clinical epidemiologists. The facilitators conducted two follow-up site visits over the 1-year intervention period. The visits occur at 3 months and 9 months. During the visits, the facilitators met the CDM team to collect information on the implementation process, discuss the challenges and document the progress in the clinics. Similar data were also collected during the intervention review workshop at 6 months. This information was analysed, and detailed feedback were then provided to each clinic. It is believed that, through this collaborative process of coaching and feedback sessions, optimal implementations tailored to the needs of each clinic could be achieved.

The control clinics continued with usual care with no additional intervention. The CDM workshop modules and intervention tools will be made available to the control clinics at the end of the study. There were no other additional resources allocated to either the intervention or the control group.

### Outcome measures

Outcome measures were obtained from both intervention and control clinics at baseline and one year after the commencement of the intervention.

#### Primary outcomes

For T2DM patients, the primary outcome is measured by the change in the proportion of patients achieving the glycaemic target of HbA1c < 6.5%.

For HPT patients without T2DM, primary outcome is measured by the change in the proportion of patients achieving the BP target of < 140/90 mmHg.

#### Secondary outcomes

The secondary outcomes were measured by changes in the proportions of patients achieving the following targets:

- BP ≤ 130/80 mmHg (for T2DM patients)
- BMI < 23 kg/m^2^
- Waist Circumference (WC) < 90 cm for men, < 80 cm for women
- Total cholesterol (TC) ≤ 4.5 mmol/L
- Triglycerides (TG) ≤ 1.7 mmol/L
- LDL-C ≤ 2.6 mmol/L
- High density lipoprotein cholesterol (HDL-C) ≥ 1.1 mmol/L

Other secondary outcome measures include:

- Change in the process of care related to the management of T2DM and HPT
- Change in the medication adherence level as measured by the 8-item Morisky Medication Adherence Scale (MMAS-8) [62]
- Change in the prescribing patterns of antihypertensive agents, oral hypoglycaemic agents, insulin usage and lipid lowering agents
- Patients’ perceptions and experiences of receiving care for chronic conditions as measured by the Patients Assessment of Chronic Illness Care (PACIC) score [63]
- Health care providers’ perceptions, attitudes, experiences and perceived barriers in implementing the EMPOWER-PAR intervention as measured by qualitative analysis
- Cost-effectiveness of the EMPOWER-PAR intervention

### Study procedures

All interviewers and investigators were trained regarding the study procedures prior to the conduct of the study to minimise variability in the method of data collection. At baseline, an interview and physical examinations were conducted. Fasting venous blood samples were obtained.

#### Demographic and anthropometric data collection

A standardised case report form (CRF) was used to collect socio-demographic information on the study subjects (age, gender, ethnicity, patient contact details, education attainment and occupation), smoking status (including the number of cigarettes smoked per day for current smokers) and other clinical information (presence of comorbidities, past medical history and family history). Data on pharmacological treatment were systematically collected from the medical records of the study subjects using CRF at baseline and at 1-year follow-up in both the intervention and the control clinics. Prescribing patterns will be analysed to assess adherence to the evidence-based prescribing recommended by the CPG.

Height and weight were measured using the Seca 769 Digital Medical Scale stadiometer. Weight was measured in light clothing, without shoes on the scale with a precision of 0.1 kg. Height was measured to 0.1 cm using the stretch stature method of the stadiometer and then converted to metres. BMI was calculated using the standard formula (weight in kg)/ (height in metres)^2^. WC was measured to the nearest 0.1 cm using non-stretchable measuring tape with the subjects standing in a relaxed position and arms at the side. The measurement was taken at the midpoint between the lower rib margin (12^th^ rib) and the iliac crest.

BP was measured twice, two minutes apart on the right arm in sitting position, using an Omron IA2 model automatic digital blood pressure monitor. Subjects were made to rest for at least 5 minutes before the measurements were taken. Each subject was seated upright with his/her right arm supported at the heart level. The mean of the first and second systolic and diastolic measurements was reported as the BP value for individual subjects.

#### Blood sampling and biochemistry profile

The baseline and outcome blood samples were analysed at the Centre for Pathology and Diagnostic Research Laboratory (CPDRL), Universiti Teknologi MARA (UiTM). Overnight fasting venous blood samples were collected following non-traumatic venepuncture. Serum separated within two hours of collection was analysed for TC, TG and HDL-C using enzymatic colourimetric reference methods on an automated analyser (Roche COBAS Integra® 400, USA). Coefficient variations (CVs) for TC, TG and HDL-C were 1.2, 3.1 and 1.0 percent, respectively, which fulfilled the Westgard and Royal College of Pathologists of Australasia CVs level. LDL-C concentration was derived by calculation using the Friedewald equation [64]. HbA1c determination was based on the turbidimetric inhibition immunoassay analysed on a similar automated analyser as that of the lipid profile with CV of 0.5, which fulfils the National Glycohaemoglobin Standardisation Program. This method has been standardised against the approved International Federation of Clinical Chemistry reference method.

### Study tools

#### Process of care questionnaire

The ‘Process of Care Questionnaire’ was used to measure the indicators of care in T2DM and HPT management. This questionnaire was developed by the investigators, and it consists of four main sections outlining 12 indicators, each with a dichotomous response (yes/no). The indicators were set based on the Malaysian CPG on the Management of T2DM [42] and HPT [43]. Data were collected from the patients’ medical records in both the intervention and the control clinics, retrospectively for two points in time (i.e. at baseline and at 1-year follow-up).

#### 8-Item Morisky Medication Adherence Scale

A previously validated Malay version of MMAS-8 [62] was used for the assessment of medication adherence levels. MMAS-8 consists of eight items with a dichotomous response (yes/no) for items 1–7 and a five-point Likert response for the last item. The total score ranges from 1 to 8, with a higher total score indicating higher medication adherence level. Scores of 1–5 indicate low adherence, 6–7 indicate moderate adherence and a score of 8 indicates high adherence level. Data collection was done by face-to-face interviews of the study patients by trained researchers. Data were collected at two points in time (i.e. at baseline and at 1-year follow-up).

#### Patients Assessment of Chronic Illness Care (PACIC) questionnaire

The quantitative measurement of patients’ perceptions and experiences of receiving care for chronic conditions was made using the PACIC questionnaire [63], which was translated into Malay language, cross-culturally adapted and validated by the investigators prior to the commencement of the study. PACIC is a 20-item patient self-report instrument to assess the extent to which patients with chronic disease receive care that aligns with the CCM, measuring care that is patient-centred, proactive and planned and which includes collaborative goal setting, problem-solving and follow-up support [63].

### Qualitative data collection procedure

A phenomenological qualitative approach using focus group discussions [65] was taken to explore health care providers’ perceptions, attitudes and perceived barriers in implementing the EMPOWER-PAR intervention at their facilities. Purposive sampling was employed, whereby the CDM team members involved in the study were invited to participate. They were then divided into three groups consisting of an FMS group, a medical officers group and an allied health care personnel group. The allied health care personnel group consisted of staff nurses, assistant medical officers, pharmacists and dieticians. A total of six groups were interviewed using a semi-structured focus group topic guide focussing on their perceptions, attitudes and perceived barriers towards implementing the EMPOWER-PAR intervention at their facilities. The discussions were audio-recorded and transcribed verbatim, systematically arranged and managed by NVivo version 10 software. Thematic analysis is used to analyse the interview transcripts. Two researchers analyse the data independently.

### Cost-effectiveness analysis

Cost-effectiveness analysis was performed from the health care providers’ perspective. The cost and outcome of the intervention program was discounted at 3% as the study time horizon is two years [66]. Resources consumed in the health care sector would include patient treatment and the EMPOWER-PAR intervention cost. Data on patient treatment cost were collected using a standardised data collection form and were valued using a bottom-up approach whereby the frequency of utilisation for each patient was multiplied by the specific charge/price. As the intervention was expected to change the consultation practice in primary care clinics, the unit cost for outpatient visits was estimated using a micro-costing approach, whereby the duration of time that a patient spent on each identified activity in an outpatient visit was measured using the time-motion technique and valued based on the local centre price. The cost of the intervention (including the CDM training workshop, a module for the trainer, Global CV Risks Self-Management Booklet for the patient and continuous support from the facilitators) was estimated using a mix of top-down and bottom-up approaches. The average total cost per patient of the intervention and control group was arithmetically expressed as follows:


The cost was expressed in 2012 Malaysian Ringgit (MYR) (USD 1 = MYR 3.30). If the intervention group was statistically more expensive and effective than usual care group, then the incremental cost effectiveness ratio was calculated as the difference between the costs for each group divided by the difference in effectiveness. The threshold of MYR 31,195 was used to determine the cost-effectiveness of the intervention [67].

### Data management

Patients’ CRF were sent to the Clinical Research Centre, and data were entered into an Excel spreadsheet. Each record was given a unique identifier consisting of a single digit for the sites and triple digits for the subjects. Data on laboratory findings were obtained from the CPDRL, UiTM in softcopy format and then merged with these records using the unique identifiers. Data cleaning was done to manage outliers, missing values and inconsistencies. The clean dataset labelled as analysis dataset was exported into SPSS and will be made available for reporting the baseline findings. It will be merged with the final visit dataset for definitive analysis.

### Sample size calculation

The sample size calculation was conducted based on the randomised clustered trial design using PASS software (Copyright (c) 2009 by Dr Jerry L. Hintze, All Rights Reserved).

#### Sample calculation for T2DM patients

A sample size of 626 (313 in each arm) was obtained by sampling 10 clusters (5 intervention vs 5 control) with 63 subjects from each cluster to achieve 91% power to detect 25% difference in the proportion of subjects achieving target HbA1c < 6.5% from baseline and between the intervention and control groups. The test statistic used was the two-sided Z-test (unpooled). The significance level of the test is 0.05. Therefore, after allowing for 25% dropout rate, this study aimed to recruit a total sample of 836 T2DM patients at baseline (i.e. 418 in each arm and 84 from each clinic).

#### Sample calculation for HPT patients without T2DM

A sample size of 438 (219 in each arm) was obtained by sampling 10 clusters (5 intervention vs 5 control) with 44 subjects from each cluster to achieve 88% power to detect 25% difference in the proportion of subjects achieving target BP < 140/90 mmHg from baseline and between the intervention and control groups. The test statistic used was the two-sided Z-test (unpooled). The significance level of the test is 0.05. Therefore, after allowing for 25% dropout rate, this study aimed to recruit a total sample of 584 HPT patients at baseline (i.e. 292 in each arm and 58 from each clinic).

### Statistical analysis

The statistical analysis plan to test the primary hypotheses of the study is described. Continuous variables will be described by summary statistics (means and standard deviations) for normally distributed variables. If the distribution is not normal, median with inter-quartile range will be reported instead. Other descriptive statistics, such as minimum and maximum values will be reported when necessary. Categorical (nominal/ordinal) variables will be described by frequencies with percentages. All outcomes are treated as categorical variables (e.g. proportions of patients achieving targets for HbA1c and BP). To assess the differences in the proportions of patients who are able to reach the treatment goals by their intervention strategy, a generalized estimation equation model that adjusts for clustering effects will be used. The model will be an independent working model. Treatment effects will be obtained using the estimated marginal means and differences will be tested using the Wald chi-square tests. The power of the study will be recalculated based on the effect size and after taking into account the clustering effect of the study design. An intention-to-treat analysis will be conducted, and *P-values* of less than 0.05 will be considered significant. All analyses will be carried out using SPSS (IBM Corp. Released 2011 IBM SPSS Statistics for Windows, Version 20.0. Armonk, NY: IBM Corp.).

### Ethical considerations

The Ethics committee of UiTM and the Medical Research Ethics Committee (MREC) of the Ministry of Health (MOH) approved the study protocol. Permission from the Family Health Development Division (FHDD) of the MOH and the respective Health District Offices was also obtained prior to the conduct of the study. The study was conducted in accordance with the Declaration of Helsinki and Good Clinical Practice (GCP) requirements [68]. Patient information sheets were distributed, and informed consent was obtained from all participants prior to their study enrolment. For participants who were unable to read, the content of the consent form was read aloud to them, and a copy of the patient information sheet was given to their next of kin with additional explanation given if needed. Confidentiality of personal information was ensured at all times. Subject enrolment was done by the investigators and not the subjects’ attending doctors to reduce subjects’ perceived coercion to participate in the study. Subjects were informed of any immediate results obtained from the study that might affect their care or health.

## Discussion

The EMPOWER-PAR is the first pragmatic randomised controlled trial of multifaceted chronic disease management strategies conducted in the Malaysian public primary care setting. It is expected to yield important new evidence on the improvements of T2DM and HPT clinical outcomes, the two most prevalent chronic diseases being managed in the public primary care sector. The EMPOWER-PAR hypothesised that patients’ clinical outcomes, namely HbA1c, BP, fasting serum lipid, BMI, WC, medication adherence and their perception and experience of receiving chronic disease care would improve with the EMPOWER-PAR intervention. It also hypothesised that improvements would occur at the primary care provider level in terms of the process of care, prescribing pattern and their perceptions, attitudes and experiences regarding the EMPOWER-PAR intervention. This study will also provide a cost-effectiveness analysis of conducting a multifaceted intervention using readily available resources in a resource-constrained setting.

The EMPOWER-PAR is also unique in the sense that it utilised a pragmatic cluster randomised trial design, which is expected to measure the degree of beneficial effect of the intervention in real life clinical practice. In pragmatic trials, a balance between external validity (generalizability of the results) and internal validity (reliability or accuracy of the results) needs to be achieved [39]. The pragmatic trial seeks to maximize external validity to ensure that the results can be generalised [39]. Therefore, the EMPOWER-PAR intervention ensured that it pragmatically utilised resources that are readily available within the system. These include strengthening the roles of allied health personnel in the CDM team, enhancing their skills to support patients’ self-management and reinforcing the utilisation of CPG to support evidence-based decision making. Although the primary outcomes for EMPOWER-PAR were measured at the patients’ level, its pragmatic design required that the intervention be delivered at the primary care providers’ level. This was done to maximise the sustainability of the intervention even after the trial ends. Some flexibility on the implementation of the intervention programme was also allowed in line with the needs and constraints of the clinics. The PAR approach [25] was therefore adopted in designing and implementing the EMPOWER-PAR intervention, where the CDM team were empowered to choose between actions within their constraints to improve their patients’ health outcomes. This process required the active participation of the CDM team from each clinic to design, propose and implement the intervention program. However, monitoring the intervention and ensuring its implementation may pose a great challenge in a pragmatic trial. Constraints within different clinics, such as high staff turnover, high workload and limited consultation time, may influence the ability of CDM team to implement and sustain the intervention. Therefore, if the EMPOWER-PAR intervention is shown to have a significant beneficial effect, it would prove not only that it can work, but also that it does work and may be sustainable in real life.

Ultimately, the results from this study will provide objective evidence of the effectiveness and cost-effectiveness of a multifaceted intervention based on the CCM in a resource-constrained public primary care setting. If proven effective, the results may be generalisable to other Malaysian public primary clinics that share the same characteristics, and the intervention would probably be inexpensive to replicate. It is hoped that the objective evidence from EMPOWER-PAR will provide a platform to instigate much needed change to the primary health care system in Malaysia.

### Trial status

Site feasibility assessment and recruitment were conducted in January–May 2012. Patient recruitment and baseline data collection were conducted in June–December 2012. The EMPOWER-PAR intervention was delivered in January–December 2013. Outcome data were collected in January–June 2014. At the time of submission of the manuscript, data cleaning and analysis has just started.
